# PLGA Carriers for Controlled Release of Levofloxacin in Anti-Tuberculosis Therapy

**DOI:** 10.3390/pharmaceutics14061275

**Published:** 2022-06-15

**Authors:** Evgeny N. Antonov, Sofya N. Andreevskaya, Irina V. Bocharova, Sergei E. Bogorodsky, Larisa I. Krotova, Elena E. Larionova, Alexandra O. Mariyanats, Gennady V. Mishakov, Tatiana G. Smirnova, Larisa N. Chernousova, Vladimir K. Popov

**Affiliations:** 1 Institute of Photon Technologies, Federal Scientific Research Centre “Crystallography and Photonics” of Russian Academy of Sciences, 108840 Moscow, Russia; e_n_antonov@mail.ru (E.N.A.); bogens2@mail.ru (S.E.B.); krollar@yandex.ru (L.I.K.); amariyanac@mail.ru (A.O.M.); mgennadii@mail.ru (G.V.M.); 2 Central Tuberculosis Research Institute, 107564 Moscow, Russia; andsofia@mail.ru (S.N.A.); 3595598@mail.ru (I.V.B.); e.larionova@ctri.ru (E.E.L.); s_tatka@mail.ru (T.G.S.); lchernousova@mail.ru (L.N.C.)

**Keywords:** anti-tuberculosis drugs, sustained release formulations, levofloxacin, PLGA microparticles and scaffolds, supercritical fluid technologies

## Abstract

Levofloxacin (LFX) is a highly effective anti-tuberculosis drug with a pronounced bactericidal activity against *Mycobacterium tuberculosis* (*Mtb*). In this work, an “organic solvent-free” approach has been used for the development of polylactic-co-glycolic acid (PLGA) microparticles and scaffolds containing LFX at a therapeutically significant concentration, providing for its sustained release. To achieve the target, both nonpolar supercritical carbon dioxide and polar supercritical trifluoromethane have been used. By changing the composition, surface morphology, size, and internal structure of the polymer carriers, one can control the kinetics of the LFX release into phosphate buffered saline solutions and physiological media, providing for its acceptable burst and desirable concentration in the prolonged phase. The biocompatibility and bactericidal efficacy of PLGA/LFX carriers assessed both in vitro (against *Mtb* phagocytosed by macrophages) and in vivo (against inbred BALB/c mice aerogenically infected with *Mtb*) demonstrated their anti-tuberculosis activity comparable with that of the standard daily intragastric levofloxacin administration. These results make it possible to consider the developed compositions as a promising candidate for anti-tuberculosis control release formulations providing for the further evaluation of their activity against *Mtb* and their metabolism in vivo over long periods of tuberculosis infection.

## 1. Introduction

The development of effective formulations for advanced therapeutic agent delivery providing for simultaneous maintenance of their bioactivity and suppression of adverse side effects is the major trend for modern pharmaceutical research and industry [1,2]. Today the majority of highly efficient drugs and protein-based active pharmaceutical ingredients (APIs) are marketed almost exclusively for parenteral administration [3,4], which often require both painful and inconvenient procedures on a daily injection basis, reducing the patient’s compliance. Bioresorbable composites comprising APIs dispersed in a certain way throughout non-toxic polymer particles or scaffolds of specific size, morphology, and porosity have widespread applications as depots and vehicles for sustained drug release formulations possessing significant advantages over conventional tablet or injection analogues [5]. These advantages include: (1) increased drug bioavailability along with reduced number and intensity of adverse reactions; (2) protection of API molecules against rapid degradation in the body by various enzymes and peptidases; (3) implementation of targeted delivery of these molecules to specific tissues and organs; (4) control of the API release rate from polymer matrices, ensuring prolongation of its therapeutic effect [6]. The long-term (weeks and over) maintenance of the API concentration at the necessary level in a certain localization of the body leads to a multiple reduction in the number of doses (injections) required, which automatically decreases its concentration fluctuations in the blood. All this lessens the risk of negative side effects and hence increases the patient’s compliance to the ongoing pharmacotherapy [7].

The time profile of the API release into the surrounding tissues and/or systematic blood flow depends on the chemical composition as well as the geometrical, morphological, structural, and physicochemical characteristics of the material used as the carrier. While bioresorbable polymers are used for these purposes, an important parameter in this case is the rate of their biodegradation in the body, which determines the drug release dynamics [8].

Aliphatic polyesters, namely, polymers and copolymers of lactic and glycolic acids (polylactides—PLA, polyglycolides—PGA, and polylactoglycolides—PLGA), are the most widely used polymer materials in the drug delivery systems [9]. Their controlled hydrolytic and/or enzymatic degradation provides the basis for the development of various types of bioresorbable carriers for targeted and prolonged drug release [10,11].

Conventional methods for preparing such formulations (e.g., spray drying and emulsion techniques) usually involve either substantial temperature perturbation or toxic organic solvents, which often leads to thermal and solvent-induced degradation of APIs or changes in their molecular conformations and bioactivity [12,13,14,15].

Over the last 25 years, an alternative “green” approach to controlled drug release system fabrication has been intensively developed and successfully implemented. It is based upon the micronization of APIs and their encapsulation into biodegradable polymers using supercritical fluids (mainly supercritical carbon dioxide—scCO_2_) both as a “green” solvent and a polymer plasticizer [16,17,18,19,20]. The entire process can be carried out at near ambient temperature (critical temperature and critical pressure for CO_2_ are *T*_cr_ = 31.1 °C and *P*_cr_ = 7.4 MPa, respectively) providing no solvent residues in the final product. This technique is applicable to a wide range of API, e.g., wound healing peptides, antibiotics, non-steroid analgesics, and antipsychotic drugs [21,22,23]. The unambiguous advantages of this methodology include the absence of organic solvents used over all fabrication steps and the possibility to control the API release characteristics by delicately tuning the process parameters [24].

The use of sustained drug release formulations is particularly beneficial for the treatment of diseases caused by *Mycobacterium tuberculosis* (*Mtb*). In clinical practice, antituberculosis drugs are usually used for a long period of time, and in doses close, as a rule, to the maximum tolerable. In this case, the delivery of API, with its controlled long-term release rate, directly to the infected area can significantly reduce the dosing frequency, thus leading to a more efficient and cost-effective therapy, reducing the degree of toxicity, and alleviating adverse effects [25].

The antimicrobial effect of controlled-release first-line antituberculosis drugs (e.g., rifampicin and isoniazid) encapsulated in PLGA microparticles was studied intensively in vitro and in vivo. These drug formulations showed high antituberculosis activity, prolonged effect, and reduced toxicity compared to their conventional counterparts [26,27].

Nowadays, to treat multidrug-resistant tuberculosis, the latest World Health Organization (WHO) guidelines recommend using group A drugs, which includes levofloxacin (LFX), exhibiting high bactericidal activity against *Mtb* [28,29].

LFX is a synthetic antibacterial drug of a broad spectrum of action [30]. It is highly active against gram-negative, gram-positive (including penicillin-resistant strains of Streptococcus pneumoniae), and “atypical” pathogens. LFX is recommended for treating patients suffering from community-acquired or nosocomial pneumonia and exacerbation of chronic obstructive pulmonary disease. It is characterized by having a good safety profile and tissue distribution as well as high bioavailability, making for the achievement of high antibiotic concentrations at the infection site [31].

The main objectives of our current research were the development and fabrication of sustained-release LFX systems based on PLGA microparticles and scaffolds, using environmentally friendly supercritical fluid (SCF) technologies, and biochemical characterization of these systems. The assessment of the application potential of the developed bioactive polymer structures as components of anti-tuberculosis long-acting formulations involved: (1) studies into kinetics of LFX release from these experimental API carriers into model physiological media, (2) analysis of their cytotoxicity by the MTT test using peritoneal macrophages (MPhs), and (3) evaluation of their bactericidal effectiveness in vitro against *Mtb* phagocytosed by macrophages and in vivo against *Mtb* in aerogenically infected inbred BALB/c mice.

## 2. Materials and Methods

In our work, polylactic-co-glycolic acids (PLGA) Purasorb PDLG5002 and PDLG7502 (Corbion Purac, Amsterdam, The Netherlands) with an inherent viscosity midpoint of 0.2 dL/g and a lactic-to-glycolic-acid monomer ratio of 50:50 and 75:25, respectively, were used as a raw polymeric material. Both copolymers are supplied in the form of white to light tan-colored granules (1–3 mm across) and are primarily used in biomedical and drug delivery applications [32].

Prior to the experiments, initial PLGA granules were thoroughly grinded by a rotary cryomill Model LZM-1M (OLIS, Moscow, Russia) together with solid carbon dioxide (as a cooling agent). The ground polymer powders were then sifted through the Model CB-04 (Vibrotekhnika, St. Petersburg, Russia) calibrated sieve set 50 μm × 50 μm and 100 μm × 100 μm in mesh size to produce a 50–100 μm polymer particle fraction for further use.

**Figure 1:**
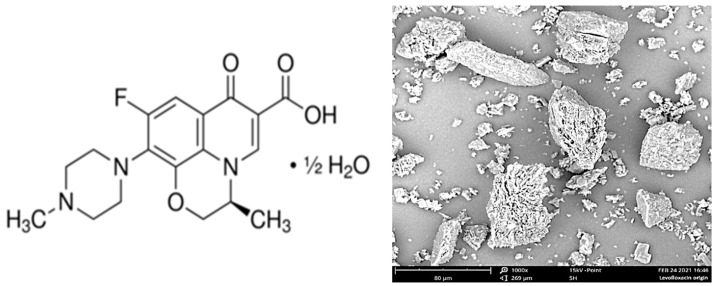
Structural formula of LFX (**left**) and scanning electron microscopy (SEM) image of its initial particles (**right**).

The initial levofloxacin substance (CAS Number: 138199-71-0; Sigma-Aldrich, St. Louis, MO, USA) (Figure 1, left) was presented as a fine powder with a mean particle size of ca. 5 to 100 μm (Figure 1, right).

Chemically pure carbon dioxide (99.98%, NIIKM Ltd., Moscow, Russia) and trifluoromethane (99.8%, Rushimprom Corp., Moscow, Russia) were used as PLGA plasticizing and foaming agents without any additional purification.

### 2.1. Fabrication of PLGA Carriers Impregnated with LFX

In our previous work [33], it was shown by the Fourier-transform infrared (FTIR) spectroscopy technique that LFX has negligible (≤10^−5^ mol %) solubility in nonpolar scCO_2_, even at a quite high temperature (60 °C) and pressure (20.0 MPa). However, the use of polar solvents, e.g., supercritical trifluoromethane (scCHF_3_, *T*_cr_ = 26.2 °C, *P*_cr_ = 4.8 MPa, dipole moment *µ* = 1.6 D), might significantly increase the solubility of polar compounds in general and, in particular, of various pharmaceutical substances [34,35]. In our recent study [36], the solubility of levofloxacin in scCHF_3_ was measured by the SCF mass-loss technique within the pressure range 10–30 MPa at a temperature from 50 to 100 °C. The solubility of LFX in scCHF_3_ at 30 MPa and a temperature of 50 °C was experimentally shown to be about an order of magnitude higher than in supercritical carbon dioxide under similar conditions—(5.6 ± 0.3) × 10^−5^ mole fractions, compared to (5.9 ± 0.3) × 10^−6^ mole fractions, respectively. Nevertheless, these values are still far from the levels sufficient to dissolve LFX in SCF in therapeutically significant concentrations.

Therefore, to achieve efficient and spatially homogeneous encapsulation of the antibiotic into bioresorbable polymer carriers (microparticles in particular), the initial pharmaceutical substance must be micronized. In our experiments, this goal was reached by precipitating LFX from an aqueous solution onto the cryogenically ground PLGA particles. To allow for 10 wt.% LFX to be loaded into the polymer structures, 100 mg of the antibiotic and 900 mg of the polymer powder were thoroughly intermixed with 3 mL distilled water, and the resultant suspension was dried for 12 h at room temperature.

**Figure 2:**
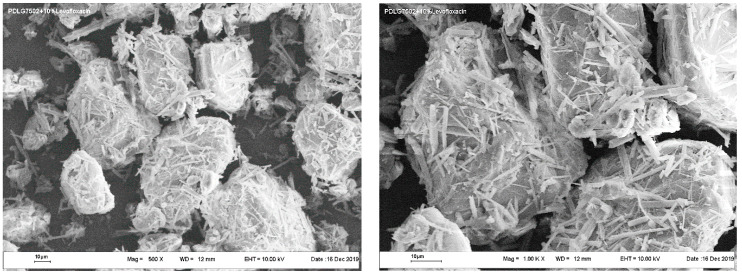
SEM images of a PLGA/LFX fine powder mixture at two different magnifications.

Typical SEM images of the PLGA microparticles coated with LFX precipitated from aqueous solution are presented in Figure 2.

The levofloxacin deposited onto polymer particles was in the form of needle-shaped microcrystals with a mean thickness of about 1 μm and a length of less than 20 μm. The finely dispersed powder of PLGA particles uniformly coated with LFX microneedles was then used to make bioactive polymer scaffolds by the SCF techniques.

In our work, biodegradable polymer scaffolds comprising a pharmaceutical substance of certain concentration were produced by the SCF plasticization and swelling of PLGA/LFX mixtures in scCO_2_ (or scCHF_3_), followed by the formation of highly porous solid polymer scaffolds upon carbon dioxide (or trifluoromethane) pressure release [37,38]. This technique relies on the ability of SCF to plasticize amorphous (or semicrystalline) polymer materials used for the encapsulation of drugs by diffusing into macromolecule chains, thus detangling them and thereby lowering their glass transition temperature (*T*_g_) [39]. The degree of polymer plasticization in this case depends on the SCF content [40].

A polymer in a gel-like (or plasticized) state can easily be impregnated with various drugs (either soluble in SCF or solid, but generally micronized) by simple mechanical or ultrasonic agitation at moderate temperatures (≤40 °C) and pressures (ca. 10 MPa) without using any organic solvents. This circumstance is crucial where thermally and/or chemically labile APIs are used.

In our experiments, a preliminarily prepared mixture of fine polymer powders with 10 wt.% of LFX deposited on them was loaded into cylindrical Teflon molds. These molds consisted of a set of 5 mm thick Teflon discs, each with 12 cylindrical holes 5 mm in diameter. The discs were crimped on both sides with solid Teflon sealing gaskets. A set of 5 discs used for the fabrication of the required number (60) of samples was crimped from the outside with a stainless-steel clamp.

The complete molds were placed in a high-pressure chamber allowing for a gradual increase in pressure and temperature, which was then sealed, purged, and filled with carbon dioxide (or trifluoromethane). The samples in the molds were processed at a pressure of 10.0 MPa and temperature of 40 °C for 1 h. Upon completion, the CO_2_ (or CHF_3_) pressure was reduced to the atmospheric value for 15 min. The samples were removed from the molds within 12 h after the end of the process. This time was enough for all the carbon dioxide or trifluoromethane to escape from the polymer structure, which then underwent final solidification.

PLGA particles impregnated with 10 wt.% of levofloxacin were produced by two different SCF techniques: cryomilling of bioactive PLGA/LFX scaffolds, fabricated by the above-mentioned method, followed by their appropriate sieving [41] and the PGSS (Particles from Gas Saturated Solutions) technique [42,43].

The PGSS technique seems to be the most promising SCF approach for the fabrication of biodegradable polymeric controlled drug-release formulations. It consists in dissolving or dispersing a fine API phase in a polymer plasticized with a subcritical or supercritical fluid, followed by its spraying through a nozzle into a low-pressure environment. The resultant mixture spraying leads to a decrease in the density and solvent power of SCF, thus inducing the formation of a supersaturated solution and a fine dispersed phase of the desired product. The plasticized material must have a reasonably low viscosity to form sprays of microparticles (ca. 50–100 μm) suitable for intramuscular injections.

In our work, the necessary bioactive particles were produced by the PGSS technique in accordance with the following algorithm. A total of 3.0 g of preliminary prepared fine PLGA/LFX powder mixture was loaded into a 75 cm^3^ high-pressure stainless steel chamber (autoclave). The chamber was sealed, heated, thermostated at 40 °C, and finally filled with SCF to a pressure of 10 MPa. At the same time, the temperature of the 900 μm dia. injection nozzle attached to the bottom of the chamber was heated to and maintained at 70 °C. The contents of the autoclave were thoroughly intermixed using a magnetic stirrer running at 150 rpm. The system was kept under these conditions for 120 min, providing for complete plasticization of the polymer and its homogeneous intermixing with LFX. After that, the resultant gel-like mixture with supercritical fluid was discharged in a pulse-periodic manner through a nozzle into a large-volume (ca. 1000 cm^3^) stainless steel receiver at atmospheric pressure.

The resultant mixture consisting of both individual microparticles and their cohered agglomerates was kept under atmospheric conditions for another 3 h to completely remove CO_2_ or trifluoromethane from the polymer particles and ensure their final solidification. Then, to destroy the agglomerates, pure nitrogen was blown into the chamber at a pressure of up to 2 MPa. The nitrogen flow captured the polymer particles and escaped from the chamber through the particle collection system comprising a glass aerodynamic cyclone and collecting vessel (Buchi, Beijing, China). As a result, the desirable finely dispersed PLGA/LFX powder fraction was obtained.

### 2.2. Optical and Scanning Electron Microscopy

The surface morphology and internal structures of PLGA/LFX microparticles and scaffolds were analyzed with an optical Bresser Advance ICQ stereoscopic microscope (Bresser, Springdale, AR, USA) equipped with a Levenhuk C510 camera (Levenhuk, Tampa, FL, USA) and also with scanning electron microscopes Phenom ProX (Phenom, Rotterdam, The Netherlands) and LEO 1450 (Karl Zeiss, Jena, Germany).

### 2.3. LFX Release Kinetics Study

The kinetics of levofloxacin release from biodegradable polymer carriers into a phosphate buffered saline solution (PBS, pH = 7.3, PanEco, Moscow, Russia) was studied using a spectrophotometer Model Cary 50 UV (Varian, Palo Alto, CA, USA) by analyzing the time variation of the integral intensity of the selected analytical LFX absorption band at 287 nm. For this purpose, the test samples were placed in glass vials containing either 200 mL PBS (for microparticles) or 100 mL PBS (for scaffolds). The vials were then sealed and placed in an incubator shaker (BiosanES-20, Rīga, Latvia) to be thermostated at 37 °C and stirred with a built-in shaker running at 90 rpm for the entire duration of the experiments (up to three months).

The concentration of LFX released into PBS for each set of five identical samples was measured every day during the first week and then 3 times per week till the end of the experiments (36 days for microparticles and 85 days for scaffolds). The pH value of the medium containing the test samples was measured once a week using a pH meter (Sartorius Basic Meter PB-11, Göttingen, Germany).

### 2.4. Cultivation of Mouse Peritoneal Macrophages and Their Infection with Mtb

Peritoneal macrophages (MPhs) were isolated from mice peritoneal exudate cells (BALB/c, ♀) 5–6 days after intraperitoneal injection of 2 mL 3% peptone (PanEco, Moscow, Russia). The MPhs were separated from non-macrophage cells through their adhesion on plastic Petri dishes, the free cells remaining in the medium being then removed by repeated washing. The adhered macrophages were transferred from the monolayer into suspension with the Versene solution (Sigma-Aldrich, St. Louis, MO, USA). The isolated macrophages were counted with a hemocytometer (LOMO, St. Petersburg, Russia). The suspension of peritoneal macrophages (50 × 10^3^ per well) was re-adhered in the 96-well plate in RPMI1640 medium (HyClone, Logan, UT, USA) with 5% FCS (HyClone, Logan, UT, USA) without antibiotics.

The H37Rv *Mtb* strain was originally obtained from ATCC (Mycobacterium tuberculosis TMC 102). Mycobacteria were passaged through C57Bl/6 mice to increase their virulence and were then stored in the Department of Immunology of the Central Tuberculosis Research Institute (CTRI, Moscow, Russia).

To infect MPhs with *Mtb*, the *Mtb* cells were added to the MPh monolayers in the ratio 1:5 (ca. 2.5 × 10^5^ CFU *Mtb* per well).

### 2.5. Cytotoxicity Testing

The cytotoxicity of the experimental samples was assessed using a suspension of pure (LFX-free) PLGA microparticles in the full RPMI1640 medium (used as a positive control), which was sequentially added to intact MPhs at concentrations of 12.5 μg/mL, 50 μg/mL, 200 μg/mL, and 400 μg/mL. Bioactive PLGA/LFX microparticles in the full RPMI1640 medium were applied at concentrations of 1.25 μg/mL, 2.5 μg/mL, and 5 μg/mL. Note that the intermediate concentration (2.5 μg/mL) corresponds to the minimal inhibitory concentration (MIC) of LFX for this system.

To assess the cytotoxic effect of PLGA/LFX scaffolds, they were mechanically reduced to small pieces weighing ca. 500 μg, which were then placed in a well with a monolayer of intact MPhs.

The cytotoxicity of the polymer carriers for MPhs was assessed through the release of lactate dehydrogenase from the destroyed eukaryotic cells into the medium, using the Promega’s CytoTox 96^®^ kit (Promega, Madison, WI, USA) in accordance with the instructions. The percentage of the lysed MPhs was determined by the formula

$$Lysis\left(\%\right)=\frac{OD_{test}-OD_{medium}}{OD_{total}-OD_{medium}},$$

where *OD_test_* is the optical density (OD) at 490 nm of the tested samples, *OD_total_* is the optical density at 490 nm for the wells containing MPhs completely lysed by the addition of acetic acid, and *OD_medium_* is optical density at 490 nm of the samples with the cultivation medium.

### 2.6. Bactericidal Activity Assessment

The anti-tuberculosis activity of PLGA/LFX structures was analyzed using an ex vivo model on *Mycobacterium tuberculosis* cells phagocytosed with mouse macrophages. For this purpose, after 24 h of phagocytosis of the mycobacteria by the macrophages, a test suspension of PLGA/LFX microparticles at a concentration of 1.25 μg/mL, 2.5 μg/mL, and 5 μg/mL was added to the wells.

The effect of PLGA/LFX microparticles on the *Mtb* cells (25 × 10^4^ CFU per well) cultivated in vitro in the RPMI 1640 full medium free from MPhs was studied first. After 7 days of exposure to the test compounds, the *Mtb* cells were cultured onto the Dubos agar medium (Difco Laboratories, Franklin Lakes, NJ, USA). The MPhs were preliminarily lysed with sterile deionized water. The *Mtb* cells were cultivated under the same conditions in vitro and ex vivo without LFX added. The initial *Mtb* culture was used as control. A series of 10× dilutions of the *Mtb* culture were prepared, and 20 μL of each was inoculated in triplicate on Petri dishes with the Dubos agar medium. The dishes were incubated at 37 °C and the colonies were counted on the 18th day of cultivation.

The effect PLGA/LFX structures on the infected macrophages was determined within 7 days after incubation by measuring the amount of the lactate dehydrogenase (LDH) enzyme with the Promega’s CytoTox 96^®^ kit (Promega, Madison, WI, USA) as described above.

### 2.7. In Vivo Analysis

Our in vivo study was performed using BALB/c female mice 20–22 g in body weight. The mice were infected with *Mtb* H37Rv using an inhalation exposure system (Glas-Col, Terre Haute, IN, USA). Three mice were humanely killed within one day of their aerosol infection to determine the CFU (colony-forming unit) of *Mtb* present in the lungs. It consisted of 100 CFU/lungs/mouse.

The total amount of the infected mice (*n* = 103) was divided into five groups: 1—negative control (NG, *n* = 23); 2—positive control for oral administration of 100 mg/kg of levofloxacin, 5 days weekly, 4 weeks after infection (LFX, *n* = 20); 3—for subcutaneous transplantation of pure PDLG 5002 microparticles less than 50 µm across after cryomilling in a dose of 20 mg/mouse, 4 weeks after infection (PL, *n* = 20); 4—for subcutaneous transplantation of PDLG 5002/LFX cryomilled microparticles less than 100 µm across in a dose of 20 mg/mouse, 4 weeks after infection (MP, *n* = 20); 5—for subcutaneous transplantation of 5 mm PDLG 5002/LFX scaffolds containing 10 wt.% levofloxacin, 4 weeks after infection (SC, *n* = 20).

The polymer carriers were subcutaneously transplanted to the infected animals in the withers area, using inhalation anesthesia with “Sevoran” (Abbott Laboratories Limited, Maidenhead, UK) within 4 weeks after infection. The oral administration of levofloxacin (Sigma, 100 mg/kg) was also started within 4 weeks of the animals’ infection. Levofloxacin was preliminarily dissolved in water, and 200 µL/mouse/day was administered 5 days weekly for 4 weeks.

On the 7th, 14th, 21st, and 28th day after transplantation of the polymer microparticles and scaffolds, and also oral administration of LFX, the mice of each experimental group (*n* = 5) were sacrificed. Their lungs were isolated, placed in PBS, and dispersed. Serial 10-fold dilutions of the lung homogenate were placed in Petri dishes with the Dubos agar. The efficacy of the anti-tuberculosis (TB) therapy was evaluated by counting the number of CFU *Mtb* in the lungs of the mice. The number of the *Mtb* colonies was counted within 21 days of incubation.

### 2.8. Statistical Analysis

The data were tested for normal distribution and equal variance. The differences between all experimental groups were analyzed by the Student’s *t*-test. In the case of non-normal distribution, the differences were assessed by the nonparametric Mann−Whitney U rank-sum test (SigmaPlot 11.0; Jandel Corporation, San Rafael, CA, USA). All values were expressed as the mean ± standard error (SE) of the mean. *p* < 0.05 was considered to be statistically significant.

## 3. Results and Discussion

### 3.1. PLGA Carriers Impregnated with LFX

The solubility of API in supercritical fluids at a given temperature and pressure is one of the critical parameters that determine the uniformity of the concentration distribution of the given pharmaceutical substance over the volume of an amorphous aliphatic polyester during its SCF impregnation.

In our previous studies [33,36], we have demonstrated that at 30 MPa and 50 °C, the solubility of LFX in scCO_2_ is four times that of ofloxacin (racemic mixture of L- and D-enantiomers of LFX) [44] and forty times higher in scCHF_3_ under similar conditions. It makes scCHF_3_ unambiguously preferable for encapsulating fluoroquinolones within PLGA structures using the SCF techniques.

However, to make the “picture” of our study complete and assess the effect of the LFX solubility in specific SCFs on the physicochemical and biochemical properties of the final products, we have used in our experiments both fluids.

**Figure 3:**
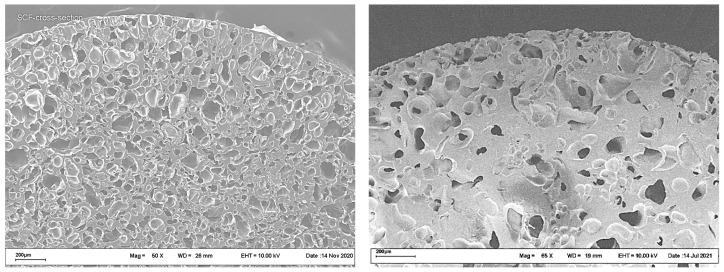
SEM images of PLGA/LFX scaffolds fabricated using scCO_2_ (**left**) and scCHF_3_ (**right**).

Typical cross-section SEM images of polyester scaffolds fabricated by the SCF plasticizing of a PLGA/LFX mixture, followed by foaming, are shown in Figure 3.

The images in Figure 3 show no significant differences in the internal structure between the samples studied. The porosity of both scaffolds was about 50%, the mean pore size being in the range 50–200 μm.

**Figure 4:**
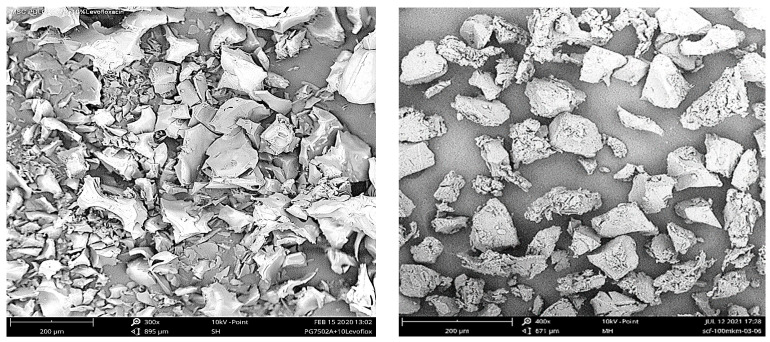
SEM images of polymer microparticles produced by means of cryogrinding from PLGA/LFX scaffolds fabricated using scCO_2_ (**left**) and scCHF_3_ (**right**).

The bioactive microparticles used less than 100 μm across and were prepared from similar scaffolds by means of mechanical cryogrinding, followed by the appropriate fractionation. The resultant microparticles had an irregular shape with a dense and well-developed surface (Figure 4).

In this case, it is also difficult to find any significant differences in the characteristic size, surface morphology, or structure between the microparticles presented.

**Figure 5:**
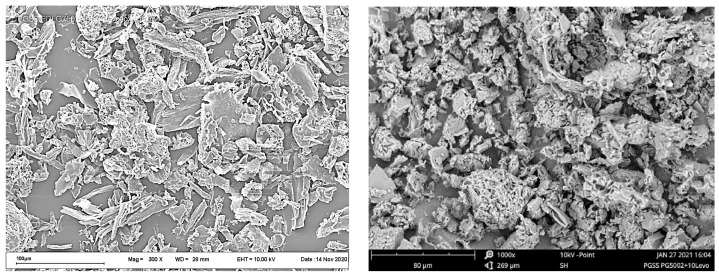
SEM images of PLGA/LFX microparticles produced by the PGSS technique using scCO_2_ (**left**) and scCHF_3_ (**right**).

In contrast, the appearance of PLGA/LFX microparticles fabricated by the PGSS technique (Figure 5) is unambiguously different from their counterparts produced by the cryogrinding of scaffolds (Figure 4). They have a rather complex structure with a well-developed surface. The particles are often seen to be interconnected by scaly fragments with a thickness of the order of a few micrometers.

However, when comparing between the polyester microparticles fabricated by the PGSS technique using scCO_2_ and scCHF_3_ (as in the case of scaffold cryogrinding), it is quite difficult to see any distinctive differences.

It is clear that the LFX microcrystals are incorporated both on and into the surface of the polymer carriers, as well as within their volume. It should also be borne in mind that part of the LFX dissolved in the SCF turns out to be uniformly distributed in the form of individual molecules and/or nanoclusters all over the volume of the polymer studied.

### 3.2. Kinetics of LFX Release into PBS

To analyze the kinetics of the release of the antibiotic from the polymer carriers, we have first plotted a calibration curve for the dependence of the integral intensity of the LFX absorption band at 287 nm on its concentration in the PBS (varied from 7.5 to 50 μg/mL). The maximal concentration that could be reached with the full yield of the LFX encapsulated in the polymer (10 wt.%) was taken as 100%.

**Figure 6:**
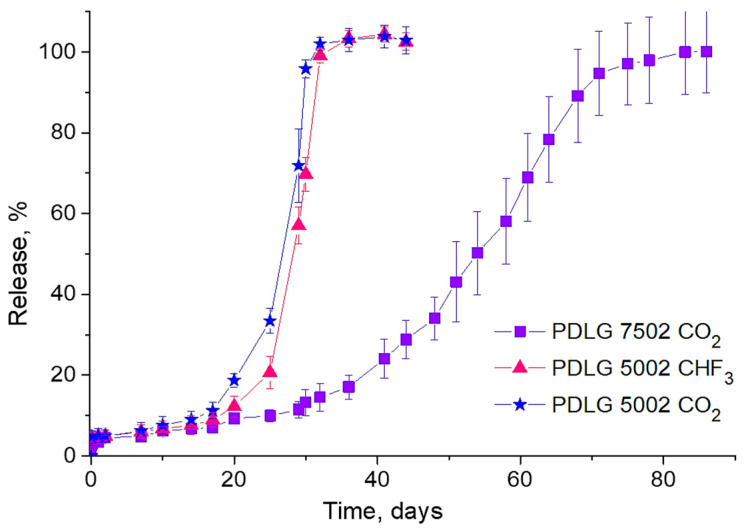
Kinetics of the LFX release into the PBS from polyester scaffolds made of various PDLG brands using different SCFs.

The results of comparative studies into the LFX release from PLGA scaffolds fabricated from PDLG 5002 and PDLG 7502 using scCO_2_ only, as well as LFX release from scaffolds formed from PDLG 5002 using two SCFs (scCO_2_ and scCHF_3_), are shown in Figure 6.

It is clearly seen that the LFX release rate from the PDLG 5002 scaffolds is almost twice that of the PDLG 7502 scaffolds, which is associated with the difference in the hydrolytic degradation rate between the polymers. At the same time, the kinetics of the LFX release from the PDLG 5002 scaffolds fabricated with two different supercritical fluids are almost identical.

This is primarily because the solubility of LFX in both scCO_2_ and scCHF_3_ under our experimental conditions is insignificant (as discussed above). Therefore, most of the LFX incorporates into the polymer in the form of recrystallized microinclusions, the majority of which are contained in its bulk rather than on the surface. This can also explain the negligible amplitude of the initial (first day) burst of the LFX concentration in the PBS for all types of scaffolds studied.

In contrast, one can see a completely different picture for the PLGA microparticles, regardless of the method through which they were obtained. In that case, a noticeable part of the LFX microcrystallites (Figure 2), comparable in size with the polymer microparticles hosting them (Figure 4 and Figure 5), will either be located on the surface of the polymer or, at least partially, coming out from its inner layers.

It is clear that the larger the fraction of LFX in these states, the greater the amplitude of the initial burst of its concentration in the PBS. On the other hand, the higher the solubility of LFX in the SCF used for its encapsulation in polyesters, the greater the proportion of individual LFX molecules and clusters of submicron size contained in PLGA microparticles. This, in turn, should lead to a decrease in the burst amplitude, as well as to the prolongation of the release of the given API associated with the hydrolysis of its host polyester structures.

**Figure 7:**
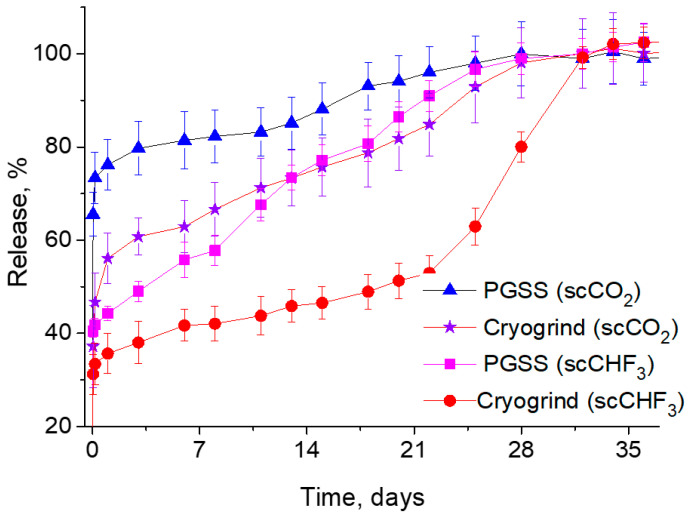
Kinetics of LFX release from polymer microparticles produced by the PGSS technique and the cryogrinding of PLGA scaffolds fabricated using scCO_2_ and scCHF_3_.

This is exactly what one can observe from the curves shown in Figure 7, which summarizes the results of our comparative studies into the dependence of the LFX concentration in the PBS on the duration of the process of its release from various PDLG5002-based microparticles produced by both the PGSS technique and the cryogrinding of polymer scaffolds fabricated using two different SCFs.

It can clearly be seen that in this case, the difference in the solubility of LFX between scCO_2_ and scCHF_3_ has a significant effect on its release kinetics. Firstly, it should be noted that the initial burst of the LFX concentration is significantly lower for the PLGA microparticles fabricated using trifluoromethane. This makes it possible to ensure that most of the LFX released into the PBS is associated with the hydrolytic degradation of the polymer carriers, rather than with the simple washout and dissolution of the LFX microcrystallites located on their surface. Secondly, the more intensive release of LFX from the PGSS microparticles in comparison with their cryogenically ground counterparts is due to their larger surface/volume ratio and, accordingly, the larger API fraction located on the surface, compared to its amount within the bulk of the polymer.

Taking into account the results described above, all subsequent in vitro and in vivo experiments were carried out on PLGA/LFX microparticles and scaffolds fabricated from PDLG5002 using scCHF_3_.

### 3.3. Cytotoxicity of PLGA-Based Carriers

**Figure 8:**
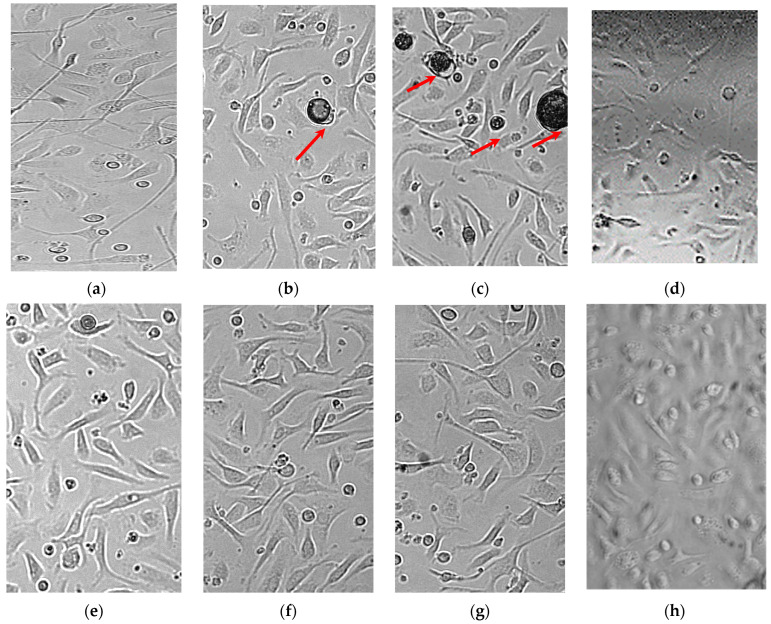
Optical images of intact murine peritoneal macrophage cultures incubated together with and without PLGA/LFX microparticles or scaffolds for 7 days. 200× magnification. The arrows in (**b**,**c**) indicate PLGA/LFX microparticles. (**a**) Spontaneous MPh lysis. (**b**) Microparticles 12.5/0. (**c**) Microparticles 200/0. (**d**) Scaffold 500 μg. (**e**) Microparticles 1.25/0.125. (**f**) Microparticles 2.5/0.25. (**g**) Microparticles 5/0.5. (**h**) MPh infected with Mtb (30–40% lysis).

The results of cytotoxicity tests for bioactive polymer microparticles and scaffolds are presented in Table 1 and Figure 8. It was shown that the extent of lysis of intact MPhs exposed to PLGA structures (both containing and free from LFX) did not differ from that of their spontaneous lysis. Higher LFX concentrations in the microparticles were not investigated to avoid the possible cytotoxic effect on the MPhs of large doses of LFX released from the microparticles into the limited volume of the MPh culture medium.

Our results indicate the absence of cytotoxic effect from the PLGA carriers (microparticles and scaffolds) even at their highest concentration tested (500 μg/well), as well as the absence of cytotoxicity from the polymer microparticles containing LFX at the doubled MIC (Figure 8g).

### 3.4. The Anti-Tuberculosis Activity of Controlled-Release LFX Formulations

The results of our studies into the anti-tuberculosis activity of PLGA/LFX microparticles with respect to *Mtb* phagocytosed by MPh in comparison with *Mtb* cultured in the Dubos medium are presented in Table 2.

An increased number of *Mtb* CFUs, relative to that of the initial *Mtb* culture, was observed in the control samples free from LFX (in vitro and ex vivo). The PLGA/LFX microparticles, at a concentration of 2.5 and 5 μg/mL, completely suppressed the growth of the mycobacteria culture concerned, and at a concentration of 1.25 μg/mL, reduced the number of *Mtb* CFU approximately by half in comparison with the initial culture.

The extent of the lysis of the infected macrophages incubated in the presence of PLGA free from LFX (control) and PLGA/LFX microparticles is presented in Table 3.

Incubation of the infected MPhs in the absence of LFX led to the lysis of 43% MPh, but their incubation in the presence of PLGA/LFX microparticles at a concentration of 1.25 µg/mL resulted in the lysis of 34% MPh (Figure 8e). Microparticles at a concentration of 2.5 and 5 μg/mL prevented the destruction of the MPhs by mycobacteria, and the lysis of these samples was comparable with the spontaneous MPh lysis (Figure 8f,g).

Thus, it was shown that the PDLG microparticles and scaffolds do not have any cytotoxic activity against MPhs, even when used at the maximum concentrations tested. The addition of PLGA/LFX microparticles to the culture of macrophages infected with *Mycobacterium tuberculosis* inhibits the growth of the pathogen, thus preventing macrophage destruction.

### 3.5. In Vivo Testing

**Figure 9:**
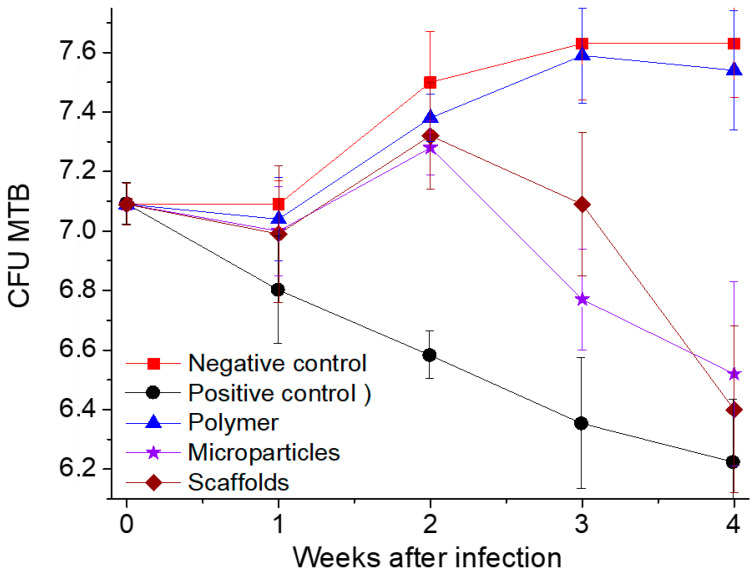
Time dependence of *Mtb* CFU/lung for different groups of mice.

The results of the in vivo studies into the anti-tuberculosis activity of our controlled-release LFX carriers on inbred BALB/c mice aerogenically infected with *Mtb* are presented in Table 4 and Figure 9.

Throughout the entire period of experimental studies, the number of *Mtb* CFUs in the lungs of mice in the negative control group (NG) and in the group of mice having LFX-free polymer transplantation (PL) steadily increased, while in the positive control group (LFX) it decreased.

Within seven days after transplantation of the polymer samples, the number of *Mtb* CFUs in the lungs of mice in all experimental groups remained almost the same and did not differ statistically (*p* ≥ 0.05).

Within two weeks after transplantation, the number of *Mtb* CFUs significantly reduced only in the mice receiving oral therapy with levofloxacin (LFX group), compared with the mice in all the other groups.

Within three weeks after transplantation of the polymeric carriers the number of *Mtb* CFUs in the lungs of the mice orally treated daily with levofloxacin continued to decrease and differed significantly from that in the other experimental groups (*p* = 0.001 between LFX vs. SC and *p* = 0.02 between LFX vs. MP).

At the same time, there was a statistically significant decrease in the *Mtb* CFUs in the groups having scaffold and microparticle transplantation (*p* = 0.01 and 0.03), compared with the amount of *Mtd* CFU in the mice of the negative control and LFX-free polymer transplantation groups. The most noticeable decrease in the number of *Mtb* CFUs was noted in the microparticle transplantation group, although there was no significant difference in the number of *Mtb* CFUs between the SC and MP groups (*p* = 0.07). The decrease in the number of *Mtb* CFUs in the lungs of mice in these groups marks the start of the levofloxacin release from the polymer microparticles and scaffolds due to their biodegradation.

Within 4 weeks after transplantation, the number of *Mtb* CFUs in the lungs of mice in the LFX, SC, and MP groups was almost the same (*p* = 0.37 at LFX vs. SC groups; *p* = 0.1 between LFX vs. MP). However, the greatest decrease in *Mtb* CFUs was observed in the mice of the SC group, which was obviously due to a significant increase in the levofloxacin concentration in their blood at that time.

These experimental results, based on the successful application of an in vivo model for chronic pulmonary tuberculosis, allow us to conclude that our biodegradable PLGA carriers impregnated with 10 wt.% levofloxacin are capable of having sufficiently high bactericidal activity. The anti-tuberculosis efficacy of the formulations developed becomes evident not immediately, but only 3–4 weeks after their implantation. Moreover, the effect of levofloxacin incorporated into the polymer (in the form of both microparticles and scaffolds) was statistically comparable with that of its daily oral administration during all the 4 weeks of the experiments.

## 4. Conclusions

The SCF plasticization of amorphous aliphatic polyesters, followed by their foaming and cryogrinding, as well as the PGSS technique using both polar (trifluoromethane) and non-polar (carbon dioxide) supercritical fluids, has been successfully used for the fabrication of bioresorbable polymer scaffolds and microparticles impregnated with anti-tuberculosis levofloxacin at a concentration of up to 10 wt.%.

The kinetics of the LFX release from PLGA carriers of various sizes into a phosphate buffered saline solution was studied. The complete LFX release from the PDLG5002 scaffolds lasted for about five weeks, while the release from the PDLG7502 ones took twice as long, this being dependent mainly on the rate of the hydrolytic degradation of the respective polymers. The initial burst of the LFX concentration in this case was very insignificant and did not exceed a few percentage points of its total amount on the first day of release.

At the same time, the LFX concentration burst for various types of PLGA/LFX microparticles varied over the first day from 35 to 75% of the API loading. Then there took place a gradual increase in the LFX concentration in the PBS, which lasted until the fifth week of the experiment, when the API release came to an end. In addition, for the PLGA microparticles fabricated using scCHF_3_, the amplitude of the initial LFX concentration burst was significantly lower compared to that of their counterparts made using scCO_2_, because most of the API distributed over their volume was released into the PBS during the course of degradation of the polymer.

Thus, by varying the composition, surface morphology, size, and internal structure of polymer carriers, one can change the rate of their hydrolytic degradation. This, in turn, makes it possible to control the polymer kinetics of the API release into physiological media to provide for an acceptable initial API concentration burst and therapeutically significant long-term API concentration.

Our ex vivo and in vivo experiments demonstrated biocompatibility of PLGA microparticles and scaffolds against eukaryotic cells and the pronounced antimycobacterial effect of these levofloxacin carriers, both against *Mtb* phagocytosed by macrophages and inbred BALB/c mice aerogenically infected with *Mtb*. The results of the in vivo assessment of the developed polymer components containing 10 wt.% LFX demonstrated their anti-tuberculosis activity, which is comparable with that of pure levofloxacin substance administered daily per OS.

All these encouraging results allow us to consider the compositions developed as promising candidates for the components of anti-tuberculosis drug release formulations, bearing in mind their further intensive study against *Mycobacterium tuberculosis* and their metabolic products in vivo over longer periods of tuberculosis infection.

## Figures and Tables

**Table 1:** Specific lysis of intact macrophages after 1 week of incubation together with the samples.

Samples	Carriers/LFX Concentration, μg/mL	MPh Lysis, %
PLGA microparticles	12.5/0	14.84 ± 0.74
	50/0	15.26 ± 1.01
	200/0	13.59 ± 1.61
	400/0	14.65 ± 0.21
	1.25/0.125	15.49 ± 0.21
	2.5/0/25	15.86 ± 0.33
	5/0.5	15.86 ± 0.33
Scaffolds	500 μg/well	15.86 ± 0.33
Control (spontaneous MPh lysis)	-	13.93 ± 0.45

**Table 2:** Growth of *Mycobacterium tuberculosis* H37Rv on the Dubos agar medium within 7 days of incubation in the presence of PLGA microparticles in vitro and ex vivo.

Samples	Carriers/LFX Concentration (μg/mL)	Dubos Medium Growth Culture (in CFU/mL)			
		In Vitro		Ex Vivo	
		Mean	SD	Mean	SD
PLGA/LFX microparticles	1.25/0.125	6.97 × 10^4^	2.08 × 10^3^	6.80 × 10^4^	3.61 × 10^3^
	2.5/0.25	No culture growth		No culture growth	
	5/0.5	No culture growth		No culture growth	
Control (free from LFX)	-	7.92 × 10^5^	4.15 × 10^4^	7.09 × 10^5^	4.80 × 10^4^
Initial *Mtb* culture	-	1.63 × 10^5^	7.64 × 10^3^		

**Table 3:** Lysis of the infected MPhs incubated in the presence of the microparticles tested.

Samples	Carriers/LFX Concentration (μg/mL)	MPh Lysis (%)
PLGA/LFX microparticles	1.25/0.125	34.76 ± 1.97
	2.5/0.25	14.63 ± 1.50
	5/0.5	13.94 ± 0.87
Control (pure PLGA)	-	43.22 ± 0.55

**Table 4:** Multiplication of *Mtb* and efficacy of the anti-tuberculosis therapy in mice for 1, 2, 3, and 4 weeks after transplantation.

Mouse Groups	Log *Mtb* CFU/Lung ± SD			
	1 Week	2 Weeks	3 Weeks	4 Weeks
1—NG	7.09 ± 0.06	7.50 ± 0.17	7.63 ± 0.19	7.63 ± 0.18
2—LFX	6.80 ± 0.17	6.58 ± 0.08	6.35 ± 0.22	6.22 ± 0.21
3—PL	7.04 ± 0.14	7.38 ± 0.08	7.59 ± 0.16	7.54 ± 0.20
4—MP	7.00 ± 0.15	7.28 ± 0.09	6.77 ± 0.17	6.52 ± 0.31
5—SC	6.99 ± 0.23	7.32 ± 0.18	7.09 ± 0.24	6.40 ± 0.8

## Data Availability

The data presented in this study are available in this article.
